# Preoperative frailty and tumor volume as determinants of primary management strategy and outcomes in petroclival meningioma patients

**DOI:** 10.3389/fsurg.2026.1865893

**Published:** 2026-08-12

**Authors:** Rommi Kashlan, Razan Faraj, Hithardhi Duggireddy, Youssef M. Zohdy, Armela Hasa, Marian Agudelo Arrieta, Estrella Barrero Ruiz, Jad Assi, Alejandra Rodas, Karen Salmeron-Moreno, Justin Maldonado, Leonardo Tariciotti, Gustavo Pradilla, Tomas Garzon-Muvdi

**Affiliations:** Department of Neurosurgery, Emory University, Atlanta, GA, United States

**Keywords:** frailty, meningioma, mFI-5, petroclival, tumor volume

## Abstract

**Objective:**

We aimed to study how patient frailty, pre-operative tumor volume, and symptom burden collectively impact primary management choice, postoperative complication rates, and outcomes in the treatment of petroclival meningiomas.

**Methods:**

We retrospectively analyzed a cohort of 73 patients diagnosed with petroclival meningiomas at Emory Healthcare from 2000 to 2022. Frailty was quantified with the 5-item modified frailty index (mFI-5). Tumor volume was measured from pre-operative MRI with 3D Slicer. Tumor-related symptoms were clustered into five domains (visual, vestibulocochlear, sensory, motor, headache) and summed into a composite Symptom Burden Score (SBS). Primary management was categorized as surgical resection versus conservative therapy (serial imaging or radiotherapy). Multivariable logistic regression evaluated predictors of treatment selection; a second model examined 30-day postoperative complications in surgical patients. Linear mixed-effects models (LMM) tracked SBS, and domain-specific generalized linear mixed-effects models (GLMM) assessed symptom resolution at 3, 6, and 12 months, controlling for both frailty and treatment approach. Sensitivity analyses dichotomized frailty (mFI-5 = 0 vs. ≥1). Significance threshold: two-sided *p* < 0.05.

**Results:**

Surgically treated tumors were larger than conservatively managed lesions (median 14.3 cm^3^ vs. 8.9 cm^3^; *p* = 0.018), and management choice varied by frailty strata (*χ*^2^ = 12.86, *p* = 0.015). In multivariable analysis, neither tumor volume (OR 1.04, 95% CI 0.97–1.11) nor mFI-5 score (OR 0.56, 95% CI 0.20–1.82) independently predicted operative selection. Thirty day complication rates increased with frailty but mFI-5 was not an independent risk factor (OR 0.51, 95% CI 0.22–1.18). Mean patient level SBS declined in both groups at 12 months (conservative −28%; surgical −8%), with a marginally significant interaction between treatment approach and frailty (*p* = 0.072). Symptom domain analysis showed significant improvement in visual and vestibulocochlear deficits irrespective of management, whereas conservative therapy was associated with poorer motor deficit recovery (*p* = 0.025).

**Conclusions:**

Preoperative tumor volume and frailty jointly influence treatment approach, yet neither metric alone dictates management decision when considered together. Perioperative risk-reduction protocols may mitigate frailty-associated morbidity. While most neurologic symptoms improve within a year, frailer patients have attenuated symptomatic improvement, in addition to domain-specific differences.

## Introduction

Petroclival meningiomas (PCMs) originate from arachnoid cap cells at the upper two thirds of the clivus at the petroclival junction, which is located medial to the internal auditory meatus and posterior to the trigeminal nerve. Their proximity to critical neurovascular structures and the brainstem renders them as one of the most complex challenges in neurosurgery, with attempts at gross total resection carrying an appreciable degree morbidity and mortality (1). Current management guidelines for these tumors includes observation through serial imaging, fractionated or stereotactic radiotherapy, and skullbase microsurgery. These clinical approaches often employed in strategic combinations in an effort to balance tumor control against neurological risk (2, 3). However, with an incidence of approximately 2% of all meningiomas and given their intricate anatomy and heterogenous clinical presentations, there remains many unanswered questions in regards to clinical management and outcomes of patients with PCMs (4). There is a need to better delineate surgical risk in these patient in order to more effectively guide treatment decisions and optimize clinical outcomes.

Recent work has therefore shifted attention toward patient specific determinants of treatment risk and post-management recovery across numerous surgical subspecialities. Frailty has emerged as a pragmatic gauge of physiologic reserve, frequently outperforming age and traditional comorbidity tallies in forecasting complications, hospital stay, and discharge disposition after surgical procedures (5–8). Frailty-based stratification has shown meningioma-specific prognostic value to predict perioperative complexity and long-term outcomes for skull base and posterior fossa lesions anatomically pertinent to the PCM (9). The 5-item modified frailty index (mFI-5), is a robust and validated comorbidity-based risk stratification tool, used to assess frailty and predict postoperative morbidity and mortality in surgical patients, including brain tumor patients (10). The mFI-5 has been increasingly used to evaluate risk in a patient-specific manner due to its simplicity, reliance on readily available clinical data, and comparable predictive performance to more complex risk stratification tools such as the mFI-11 (11, 12).

In addition, volumetric analyses of intracranial tumors have demonstrated that larger tumor volume is associated with an increase in tumor related symptom burden at presentation, which can influence treatment selection and diminish postoperative functional gains (13–16). However, there is limited research in PCM cohorts on how preoperative frailty may further influence treatment decisions and predict clinical outcomes when combined with preoperative tumor volume and symptom burden. While symptomatic tumors and larger lesions typically warrant surgical intervention, it remains unclear how preoperative frailty may impact clinical decision making and effect longitudinal outcomes in patients with PCMs (16–18). Clarifying these relationships is critical for developing risk stratified counseling and for tailoring multimodal strategies to each patient's unique physiologic and anatomic profile.

To address these gaps in knowledge, we performed a retrospective cohort study on 73 patients integrating frailty status, volumetric tumor analysis, and a domain specific Symptom Burden Score (SBS) in patients with PCMs. Frailty was quantified with the mFI-5, tumor volume was measured from preoperative MRI using the software 3D Slicer, and tumor related symptoms, including, visual deficits, vestibulocochlear deficits, sensory deficits, motor deficits, and headache, were combined into a composite SBS (19). We investigated how each factor, individually and synergistically, influenced primary management decision-making among observation, radiotherapy, and resection. In addition, we investigated the predictive capabilities of these factors on postoperative complications and clinical outcomes up to 12 months post-intervention. By coupling multivariable logistic regression with mixed effects modeling, we aimed to delineate the interplay between frailty, tumor volume, and symptom burden, providing data driven insight to refine risk stratified counseling and enhance patient-specific, multimodal management for this challenging skullbase tumor.

## Methods

### Study design and patient population

We performed a retrospective cohort study of 73 patients diagnosed with petroclival meningiomas (PCMs) who presented to Emory Healthcare between 2000 and 2022. Emory institutional review board approval was obtained with waiver of informed consent owing to minimal risk. Inclusion criteria were: (i) pathologically or radiographically confirmed PCM; (ii) availability of preoperative, contrast enhanced MRI suitable for volumetric analysis; and (iii) complete clinical documentation permitting calculation of the 5-factor modified frailty index (mFI-5) and Symptom Burden Score (SBS). Patients with recurrent tumors previously treated elsewhere or with minimal clinical data were excluded.

### Data collection

Clinical and radiographic variables were extracted from the electronic medical record. Frailty was quantified by the mFI-5, assigning one point each for functional dependence, diabetes mellitus, chronic obstructive pulmonary disease, congestive heart failure, and hypertension requiring medication. In our primary models, patients were categorized into the following frailty categories: 0 (physiologically robust), 1 (low frailty), 2 (moderate frailty), and 3+ (high frailty). Frailty data was converted to a binary variable as robust (mFI-5 = 0) or frail (mFI-5 ≥ 1) for sensitivity analyses. Tumor volume was measured on preoperative MRI using the software 3D Slicer. Volumes were recorded in cubic centimeters (cm^3^) and treated as a continuous variable. Symptom Burden Score (SBS) captured tumor-related symptoms at presentation, which included vestibulocochlear deficits (hearing loss, tinnitus, imbalance), visual deficits (diplopia, acuity loss, field deficit), sensory deficits (facial pain, numbness), motor deficits (dysphagia, dysarthria, limb weakness), and headache. One point was assigned for each symptom recorded and incidental/asymptomatic cases scored 0. Primary management was categorized as conservative (serial imaging or radiotherapy) or surgical resection. Outcome measures included: (i) 30-day postoperative complications (any medical or surgical adverse event requiring intervention) and (ii) longitudinal symptom trajectories at 3, 6, and 12-months follow-up.

### Statistical analysis

Continuous variables are summarized as mean ± standard deviation (SD) or median (interquartile range, IQR), as appropriate, and categorical variables as counts and percentages. Normality was assessed with the Shapiro–Wilk test. Tumor volume and mFI-5 across intervention groups were analyzed with oneway ANOVA or Kruskal–Wallis tests, followed by Dunn's posthoc comparisons with Bonferroni adjustment. Categorical variables were compared using Pearson *χ*^2^ or Fisher's exact tests. A multivariable logistic regression model examined predictors of undergoing surgery, with tumor volume, mFI-5 score, their interaction term, age, sex, and SBS entered *a priori*. Model fit was assessed with the Hosmer–Lemeshow test and area under the receiver operating characteristic curve. Among surgical patients, univariable and multivariable logistic regression evaluated the association of frailty (mFI-5) with 30-day complications, adjusting for age, sex, tumor volume, and operative duration. Linear mixed effects models (LMM) estimated change in continuous SBS over time, including fixed effects for time point, mFI-5, tumor volume, treatment type, and their interactions, with patient level random intercepts. Domain specific symptom resolution (vestibulocochlear, visual, sensory, motor, and headache) was analyzed using generalized linear mixed effects models (GLMM) with a logit link. All regression models were repeated using a binary frailty variable (mFI-5 = 0 vs. ≥1) to test robustness through sensitivity analysis. Statistical significance was defined as two-sided *p* < 0.05. Analyses were conducted in R version 4.3.2.

### Data presentation

Summary statistics, regression coefficients, and model diagnostics are provided in the accompanying tables (see Tables).

## Results

### Participants and descriptive statistics

There were 73 PCM patients from 2000 to 2022 included in the study. Patient demographics and clinical characteristics stratified by frailty score (mFI-5) are summarized in Table 1. Nearly half of our patient cohort was classified as physiologically robust (mFI-5 = 0; n-36, 49.3%), 23 (31.5%), 12, (16.4%), and 2 (2.7%) patients were assigned mFI-5 scores of 1 (low frailty), 2 (moderate frailty), and 3+ (high frailty) respectively. The patient cohort was predominantly female (67%; *n* = 49) and sex distribution did not vary significantly across frailty strata (*p* = 0.521). Mean patient age at initial presentation rose significantly with higher frailty, increasing from 51.5 ± 12.9 years in the physiologically robust group to 68.7 ± 10.2 years among patients with moderate frailty (*p* = 0.002).

**Table 1:** Demographic and clinical characteristics stratified by frailty score (mFI-5).

mFI-5 score	0	1	2	3+	*p*-value
*n*	36	23	12	2	
Female	20	19	9	1	0.521
Male	16	4	3	1	
Mean Age	51.53	55.22	68.7	58.5	0.002
Mean Tumor Volume	25.09	8.62	16.76	27.6	0.059
Symptomatic	25	22	9	2	0.309
Incidental	11	1	3	0	
Mean Symptom Burden Score	2.86	3.91	1.83	2	0.092

Continuous variables compared using ANOVA; categorical variables compared using Chi-square tests.

Preoperative tumor volume showed a marginally significant trend toward lower volumes in low frailty patients and higher volumes in the high frailty (*p* = 0.059). Mean preoperative tumor volumes ranged from 8.6 cm^3^ (low frailty) to 27.6 cm^3^ (high frailty). Most patients (81%) were symptomatic at presentation, and this proportion did not differ significantly by frailty stratification (*p* = 0.309). The mean baseline Symptom Burden Score (SBS) was 3.0 ± 2.3 overall, with trending significance in frailty dependent variation (*p* = 0.092).

Analysis of individual mFI-5 components revealed that hypertension requiring medication was the most prevalent contributor (43.8%), followed by diabetes mellitus (16.4%), functional dependence status with an inclusion criterion of a Karnofsky Performance Status (KPS) score of less than 80 (5.5%), congestive heart failure (4.1%), chronic obstructive pulmonary disease (1.4%) (20).

### Preoperative frailty, tumor volume and primary management choice

Preoperative tumor volume comparisons of 50 patients undergoing either conservative or surgical management are summarized in Table 2. 56% of patients underwent surgical resection, whereas the remaining 44% were managed conservatively with observation or radiotherapy. Median preoperative tumor volume differed significantly between these management groups. PCM cases selected for surgery were, on average, nearly twice as large as those managed conservatively [14.3 (IQR 7.32–26.37) cm^3^ vs. 8.9 (IQR 3.47–11.22) cm^3^; Kruskal–Wallis *p* = 0.018)].

**Table 2:** Comparison of tumor volumes between conservative and surgical management groups.

Management	*N*	Median tumor volume (cm_3_)	Q1–Q3 tumor volume		*χ*²	*p*-value
Conservative	20	8.93	3.47–11.22		NA	0.018
Surgical	30	14.26	7.32–26.37			

mFI-5 score	0	1	2	3	*χ*²	*p*-value
Conservative	6	10	9	0	12.86	0.015
Surgical	24	12	3	2		

Statistical test: Kruskal–Wallis test. Distribution of treatment strategy (Conservative vs. Surgical) across frailty levels (mFI-5). Statistical test: Chi-square test.

The distribution of treatment strategy stratified by frailty status is summarized in Table 2. The distribution of primary management choice varied significantly based on frailty status *χ*^2^(3, *N* = 66) = 12.86, *p* = 0.015). Among conservatively managed patients, only 30% were physiologically robust (mFI-5 = 0) compared with 80% in the surgical cohort. 45% of patients who were conservatively managed had moderate frailty (mFI-5 = 2) compared to 10% of surgical cases. Interestingly, no patient with high frailty (mFI-5 ≥ 3) underwent conservative management as a primary intervention; both individuals of this frailty status proceeded to surgery.

The results of a multivariable logistic regression model using preoperative tumor volume and frailty status as predictors of surgical intervention are summarized in Table 3, with sensitivity analysis summarized in Table 3. Neither frailty status (OR = 0.56, 95% CI 0.20–1.82; *p* = 0.37) nor tumor volume (OR = 1.04, 95% CI 0.97–1.11; *p* = 0.25) independently predicted the decision to operate, and their interaction was non-significant, as well (OR = 0.99, 95% CI 0.95–1.03; *p* = 0.53). To test robustness, we repeated the analysis with frailty coded as a binary variable (mFI-5 = 0 vs. ≥1). While the odds ratio was modestly strengthened, the results were unchanged with binary frailty (OR = 5.16, 95% CI 0.63–42.50; *p* = 0.13), tumor volume (OR ≈ 1.00, 95% CI 0.96–1.05; *p* = 0.89), and their interaction (OR = 1.02, 95% CI 0.94–1.11; *p* = 0.65) not reaching significance; however, frailty as a binary variable appeared to be more predictive of surgical intervention than tumor volume.

**Table 3:** Logistic regression results predicting the decision for surgical intervention based on frailty (mFI-5), tumor volume, and their interaction.

Variable	Coefficient (β)	Standard error (β)	Odds ratio	95% CI	*p*-value
mFI-5	−0.50	0.56	0.61	0.20–1.82	0.37
Tumor Volume	0.038	0.0332	1.04	0.97–1.11	0.25
mFI-5 and Tumor Volume	−0.013	0.0222	0.99	0.95–1.03	0.53

Variable (sensitivity)	Coefficient (β)	Standard error (β)	Odds ratio	95% CI	*p*-value
mFI-5 = 0 vs. mFI-5 ≥ 1	1.64	1.076	5.16	0.63–42.50	0.13
Tumor Volume	0.0033	0.025	∼1.00	0.96–1.05	0.89
Interaction	0.02	0.044	1.02	0.94–1.11	0.65

Sensitivity analysis logistic regression predicting surgery using a binary frailty classification (mFI-5 = 0 vs. mFI-5 ≥ 1). CI formula: exp[β ± 1.96 × SE_β_].

### Postoperative complications and frailty

Complication free recovery was most frequent among physiologically robust individuals, with complications recorded in 14% of mFI-5 = 0 patients, compared with 50% in the low frailty group, 67% in the moderate frailty group, and 50% in the high frailty group.

The results of a multivariable logistic regression model using frailty status as a predictor of postoperative complications are summarized in Table 4, with sensitivity analysis summarized in Table 4. When frailty was modelled as an ordinal variable, increasing frailty was a marginally significant predictor of potential postoperative complications (OR 0.51, 95% CI 0.22–1.18; *p* = 0.12). However, since an odds ratio of 1 is included in the confidence interval, despite complication rates showing a visible gradient when stratified by frailty status, postoperative complications could not reliably be independently determined by frailty. Re-estimating the model with a binary frailty definition (mFI-5 0–1 vs. ≥2) did not reach statistical significance, despite pooling moderate and high frailty statuses (OR = 1.92, 95% CI 0.27–13.63; *p* = 0.52).

**Table 4:** Logistic regression model assessing the association between frailty (mFI-5) and risk of postoperative complications.

Variable	Coefficient (β)	Standard error (β)	Odds ratio	95% CI	*p*-value
mFI-5	−0.68	0.43	0.51	0.22–1.18	0.12

Variable (sensitivity)	Coefficient (β)	Standard error (β)	Odds ratio	95% CI	*p*-value
mFI-5 (0–1) vs. mFI-5 ≥ 2	0.65	1.001	1.92	0.27–13.63	0.52

Sensitivity analysis logistic regression predicting complications post-surgery using alternative frailty categorization (mFI-5 0–1 vs. mFI-5 ≥ 2). Sensitivity analysis logistic regression predicting complications post-surgery using alternative frailty categorization (mFI-5 0–1 vs. mFI-5 ≥ 2).

### Symptom burden and frailty

**Figure 1:**
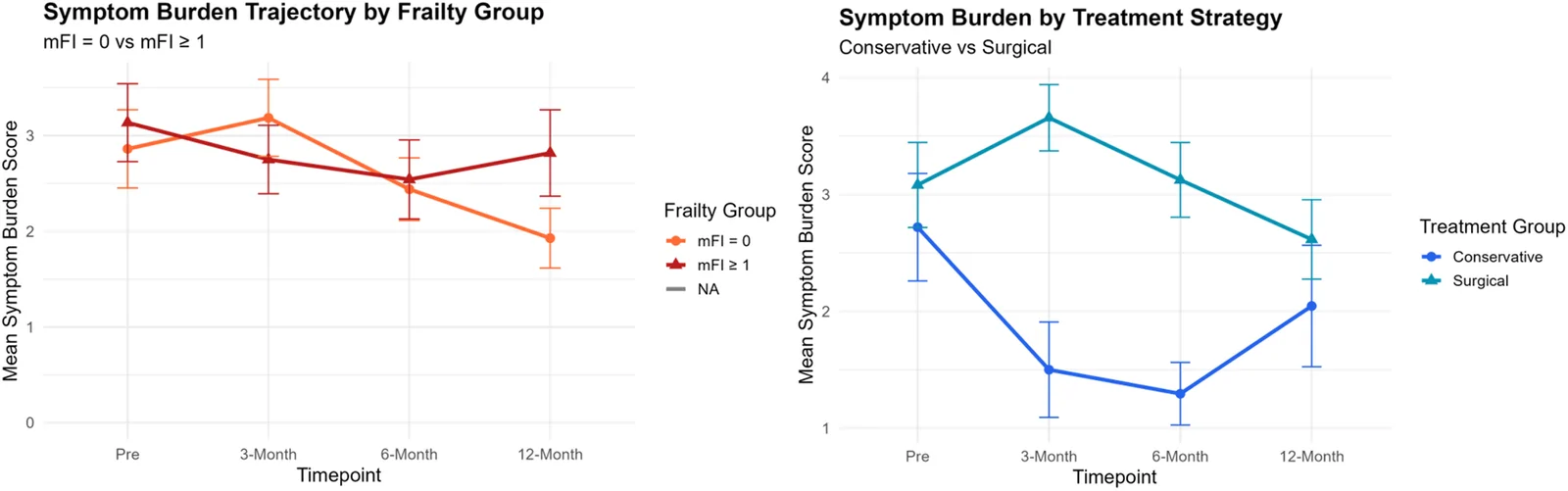
Symptom burden score trajectories by frailty group and treatment strategy. Mean Symptom Burden Score (SBS) with 95% confidence intervals at pre-treatment, 3, 6, and 12 months. *Left panel:* SBS trajectories stratified by frailty status (mFI = 0 vs. mFI ≥ 1) across all patients. *Right panel:* SBS trajectories stratified by primary management strategy (conservative vs. surgical), with conservatively managed patients showing a steeper early decline in symptom burden.

Results from a linear mixed-effects model (LMM) of longitudinal follow up over a 12-month period for symptom burden with and without the influence of frailty are summarized in Table 5. Baseline Symptom Burden Score (SBS) was higher on average in the surgical cohort (3.74 ± 2.45) than in the conservatively treated cohort (2.65 ± 2.39). By 12 months follow-up, patients from both treatment group cohorts had improvements in their SBS (Surgical: 2.63 ± 2.15, Mean Patient Level Percent Change = −8.40%; Conservative: 2.04 ± 2.38, Mean Patient Level Percent Change = −28.10%). The percent change calculated in Table 5 represents the mean of each patient's individual percent change from baseline to 12 months, rather than a percent change calculated from cohort-level means. This patient-level approach was done to avoid distortion from variability in baseline scores and more accurately reflects the clinical improvement experienced by individuals. Neither treatment approach (*p* = 0.102) nor frailty (*p* = 0.538) individually determined clinical improvement in this patient cohort; however, when the effects of treatment approach and frailty were examined together, there was a marginally significant difference in clinical outcomes (*p* = 0.072). Figure 1 illustrates this pattern, as the physiologically robust patients (mFI = 0), demonstrated a steady decline in SBS, whereas patients with some degree of frailty (mFI ≥ 1), had a plateau in the fall of their SBS, yielding a smaller net symptomatic improvement at 12 months. This trend, however, was not statistically significant as determined by our LMM (*p* = 0.538). When plotting SBS over time by treatment approach in Figure 1, conservatively managed patients had greater improvement earlier on in their follow-up period, with diminishing improvement past 3 months. Surgical patients showed a more gradual, steady decline in their SBS. However, these visual differences were not statistically significant as determined by our LMM (*p* = 0.102).

**Table 5:** Linear mixed-effects model (LMM) results for symptom burden scores at 12 months follow-up.

Group	Baseline SBS (mean ± SD)	12-month SBS (mean ± SD)	Group-level ΔSBS (mean)	Mean patient-level percent change (%)^a^	*P* (time × treatment)	*P* (time × frailty)	*P* (time × treatment × frailty
Conservative	2.65 ± 2.39	2.04 ± 2.38	−0.61	−28.10%	0.102	0.538	0.072
Surgical	3.74 ± 2.45	2.63 ± 2.15	−1.11	−8.40%			

Including interactions with frailty (mFI-5) and primary treatment choice.

a Percent change reported is the mean of each patient's individual percent change from baseline to 12 months, rather than the percent change of the group-level means.

**Figure 2:**
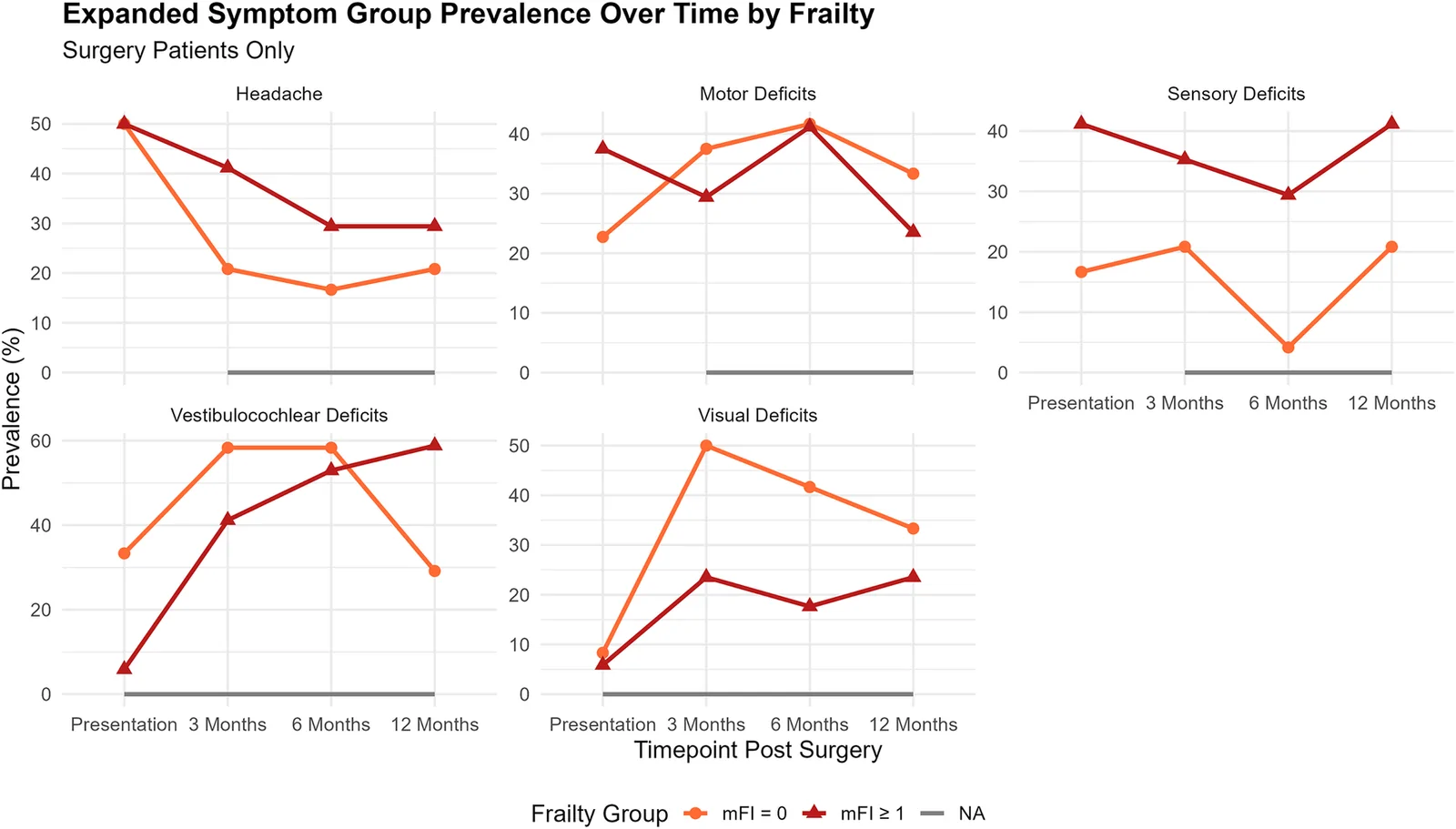
Expanded symptom group prevalence over time by frailty status in surgically managed patients. Prevalence (%) of five symptom domains (headache, motor deficits, sensory deficits, vestibulocochlear deficits, and visual deficits) at presentation and at 3, 6, and 12 months post-surgery, stratified by frailty group (mFI = 0 vs. mFI ≥ 1). Each panel represents one symptom domain.

The results from generalized linear mixed-effects modeling (GLMM) assessing symptom resolution over time across different symptom domains, controlling for frailty status and treatment approach (conservative vs. surgical) are summarized in Table 6, with prevalence plots in Figure 2 corresponding to GLMM results. Visual deficits prevalence fell markedly over 12 months (*p* < 0.001), and improvement was independent of frailty status (*p* = 0.15) or treatment approach (*p* = 0.99). Vestibulocochlear deficient prevalence increased transiently in frail patients (mFI ≥ 1), whereas physiologically robust patients (mFI = 0) had a transient decrease in prevalence after 6 months. Our GLMM demonstrated a statistically significant improvement overall in vestibulocochlear deficits in our cohort over time (*p* < 0.001), independent of frailty status, but with marginal significance based on treatment approach, favoring surgery for better improvement (*p* = 0.08). Improvements in sensory deficits seemed to peak at 6 months in both frail and non-frail patients (*p* = 0.008), with a rebound in symptom prevalence after this timepoint in both groups. This trend was independent of frailty status (*p* = 0.95) and treatment approach (*p* = 0.54). There was minimal change in the prevalence of motor deficits over 12 months. Conservative treatment was significantly associated with poorer motor outcomes (*p* = 0.025). Prevalence of headache declined sharply until 6 months and plateaued thereafter in both frail and non-frail patient cohorts. Improvement in headache was marginally associated with frailty status (0.12) and treatment approach, favoring surgery (0.079).

**Table 6:** Generalized linear mixed-effects models (GLMM) assessing symptom resolution over time across different symptom domains (visual, vestibulocochlear, sensory, motor, headache), controlling for frailty (mFI-5) and treatment approach (conservative vs. surgical).^a^

Symptom domain	Model term	Estimate (β)	Standard error	Z-value	*p*-value	95% CI lower	95% CI upper
Visual	Month 3	3.67	0.89	4.13	<0.001	1.93	5.41
	Month 6	3.11	0.86	3.63	<0.001	1.43	4.78
	Month 12	3.11	0.86	3.63	<0.001	1.43	4.78
	Frailty	−0.83	0.57	−1.45	0.15	−1.95	0.29
	Treatment	−0.01	1.01	−0.01	0.99	−1.99	1.98
Vestibulocochlear	Month 3	1.77	0.53	3.32	<0.001	0.73	2.82
	Month 6	2.06	0.54	3.82	<0.001	1.00	3.12
	Month 12	1.87	0.54	3.49	<0.001	0.82	2.92
	Frailty	−0.11	0.37	−0.30	0.76	−0.83	0.61
	Treatment	−1.15	0.66	−1.75	0.08	−2.44	0.14
Sensory Deficits	Month 3	−0.83	0.64	−1.31	0.19	−2.08	0.42
	Month 6	−1.99	0.75	−2.66	0.0079	−3.47	−0.52
	Month 12	−0.83	0.64	−1.31	0.19	−2.08	0.42
	Frailty	0.05	0.75	0.06	0.95	−1.42	1.52
	Treatment	−0.80	1.32	−0.61	0.54	−3.38	1.78
Motor deficits	Month 3	−0.15	0.49	−0.30	0.76	−1.11	0.82
	Month 6	0.08	0.49	0.16	0.88	−0.88	1.03
	Month 12	−0.15	0.49	−0.30	0.76	−1.11	0.82
	Frailty	−0.16	0.35	−0.45	0.65	−0.84	0.52
	Treatment	−1.47	0.66	−2.24	0.025	−2.75	−0.18
Headache	Month 3	−2.03	0.53	−3.79	<0.001	−3.07	−0.98
	Month 6	−2.32	0.56	−4.14	<0.001	−3.42	−1.22
	Month 12	−1.40	0.49	−2.85	0.0044	−2.36	−0.44
	Frailty	0.60	0.38	1.56	0.12	−0.15	1.35
	Treatment	−1.21	0.69	−1.75	0.079	−2.56	0.14

a β > 0: higher log-odds of the symptom being present (worse or no improvement); β < 0: lower log-odds of the symptom being present (better; greater likelihood of resolution). Reference Variable for Treatment: Conservative Treatment.

## Discussion

### Key results

Our retrospective cohort study analyzing 73 patients diagnosed with PCM provides a multifaceted view of clinical management by examining frailty, preoperative tumor volume, and symptom burden collectively as determinants of treatment selection and outcomes. Baseline analyses confirmed that surgically treated tumors were substantially larger than those managed conservatively and that treatment allocation differed across frailty strata. However, the degree to which tumor anatomy and preoperative frailty were weighed in clinical decision making remained unclear. Multivariate analysis demonstrated that once frailty status and tumor volume were incorporated into the same model, neither retained independent predictive value for primary management approach, implying that neurosurgeons integrate anatomic burden and physiological reserve jointly rather than applying either criterion in isolation (21, 22).

Despite a visible gradient in unadjusted postoperative complication rates rising with frailty status, frailty did not emerge as a significant predictor postoperative morbidity. Both ordinal and dichotomous logistic regression modelling with frailty as a predictor of postoperative complication rates resulted non-significant odds ratios with wide confidence intervals, suggesting that safeguards, such as peri-operative optimization protocols, may be in place to mitigate frailty-related risk (23).

Symptom Burden Scores (SBS) declined significantly over the 12-month follow-up period in both treatment arms. This result was independent of treatment approach and frailty alone, when their effects were evaluated separately. When the effects of frailty and treatment were combined in our LMM, there was a marginally significant difference in the overall symptomatic improvement. This suggests that patient frailty may differentially modulate recovery patients in surgically treated patients versus conservatively treated patients. Symptom domain-specific modelling refined these observations. Visual and vestibulocochlear deficits improved consistently regardless of frailty status or treatment approach, whereas conservative management was associated with inferior motor deficit improvement compared to surgical management. There was a marginal delay in vestibulocochlear deficits improvement in conservatively managed patients versus surgically managed patients, but this was not significant. Headache showed the most pronounced and rapid decline across all timepoints, independent of frailty, highlighting its responsiveness to both surgical resection and nonoperative tumor control. Sensory deficits followed an intermediate course and were independent of frailty status or treatment approach, with improvement reaching significance at 6 months and subsequently plateauing. This suggests that partial but durable relief is achievable even without further change at one year. Collectively, these mixed-model results demonstrate that most PCM-related neurologic symptoms improve substantially within 6 to 12 months, but frailty attenuates the magnitude of global symptom relief.

### Interpretation and clinical implications

These findings suggest that treatment selection for PCMs is governed by a dynamic balance between anatomic burden and physiological reserve rather than by either factor alone. Tumor volume remains the most intuitive factor for surgical decision-making; nevertheless, its influence is attenuated in the presence of diminished physiological reserve, determined by a patient's frailty status, indicating potential for an increase in postoperative complications or limited postoperative improvement in symptom burden. Conversely, our results indicate that low frailty does not automatically mandate surgical resection, even for a tumor volume that may meet an operative threshold. Even more interestingly, 2 patients who were assigned a 3+ frailty score underwent surgery, which may be due to high baseline SBS need for faster relief. This multifactorial interdependence reinforces the importance of an individualized decision matrix in which volumetric thresholds are modulated by comorbidity-based frailty assessment, with additional consideration of baseline symptomatic burden (16).

The absence of an independent frailty signal for post-operative complications, despite our data showing numerically higher complication rates in frailer patients, indicates that appropriate peri-operative optimization protocols, such as prehabilitation and enhanced recovery after surgery (ERAS), can serve to reduce risk traditionally ascribed to patients with higher pre-operative morbidity (23). With risk mitigation strategies in place, from a clinical standpoint, frailty may be viewed as a modifiable risk factor, rather than an absolute contraindication for surgical intervention. High frailty patients with compelling neurological indications for surgery may still be considered operative candidates, provided that peri-operative optimization protocols are rigorously applied and the physiological cost-benefits of a surgical procedure are clearly communicated to the patients.

Symptom-trajectory modelling offers additional nuance for patient counseling. Our data demonstrating reliable improvement of visual and vestibulocochlear symptoms, regardless of treatment modality, suggests that these symptom domains may be driven by tumor mass effect and may respond with adequate control of tumor growth or volume reduction (24, 25). However, follow-up MRI volumetric analysis would be needed to confirm this. In contrast, motor recovery appears to favor surgical resection, and clinicians should caution conservatively managed patients that gait or swallowing deficits may persist or worsen (26, 27). Headache demonstrated the greatest and most rapid improvement, making it a reassuring marker when present in isolation for a positive outcome. Sensory symptoms improved modestly and plateaued, indicating that treatment planning should involve communication to the patient that partial relief rather than complete resolution is the realistic expectation in this domain (28).

Taken together, these findings provide a quantitative context for the advocacy of a multidimensional clinical decision-making approach that integrates tumor size, frailty status, and domain specific symptom priorities. For example, a robust patient with a large tumor and disabling motor deficit may reasonably elect surgery despite baseline comorbidity risk, whereas a frail individual with stable visual symptoms and a small lesion may derive equal or greater benefit from observation or radiotherapy. The model also underscores the value of longitudinal, symptom specific follow-up, as discrete domains appear to evolve at different rates. Targeted rehabilitation (e.g., vestibular therapy, swallowing exercises) may be timed to coincide with each domain's window of maximal recovery potential (29, 30). The attenuation of symptom relief in frailer patients underscores the importance of prehabilitation and rigorous postoperative rehabilitation to maximize neurologic gains (31).

### Limitations

The study's retrospective design and single center setting may introduce selection and referral bias, limiting generalizability. The small number of patients in the highest frailty group reduced statistical power to detect modest effect sizes in this subgroup and widened the confidence intervals in our multivariable models. Surgical variables that may influence operative complexity and outcomes, such as tumor grade, surgical approach, extent of resection, and individual surgeon experience, were not captured. Symptom Burden Score, although pragmatic, relies on clinical documentation and therefore may underrepresent subtle sensory or cognitive disturbances. Follow up was confined to 12 months; longer horizons are necessary to evaluate durability of tumor control, late cranial nerve morbidity, and radiation related sequelae. Finally, the conservative management variable encompassed both serial imaging and primary radiotherapy, a heterogeneity that may have diluted treatment specific effects.

### Future directions

Future work should prioritize multi-institution, prospective databases that incorporate automated volumetric assessment, frailty biomarkers (e.g., inflammatory markers), and standardized patient reported outcome measures, such as Quality of Life (QoL). This approach would enable adequately powered analyses of high frailty subgroups with additional correlates and permit greater generalizability. Propensity matched or randomized comparisons of surgery versus stereotactic or fractionated radiotherapy in moderately frail patients would clarify the tradeoffs between tumor control and outcomes suggested by our domain specific models. Longer (≥5year) follow-up is essential to capture late recurrences, radiation toxicity, and sustained quality of life trajectories. Integration of radiomic features and machine learning algorithms may further refine patient specific risk prediction, while dedicated studies of targeted rehabilitation could optimize recovery windows identified in this analysis.

## Conclusions

In this retrospective cohort study of 73 patients with PCMs, tumor volume and frailty were both associated with primary management choice, yet neither independently dictated the decision to operate when considered together. While frailty modulates the degree of postoperative symptomatic improvement, post-surgical complications were not independently driven by frailty in this cohort. These results support a nuanced, multidimensional approach to PCM management that balances anatomic burden, physiological reserve, and symptom profile to optimize patient outcomes.

## Data Availability

The datasets presented in this article are not readily available because this is proprietary dataset. Requests to access the datasets should be directed to rommi.kashlan@emory.edu.
